# Supplementary material for: Holistic Photoprotection, Broad Spectrum (UVA-UVB), and Biological Effective Protection Factors (BEPFs) from Baccharis antioquensis Hydrolysates Polyphenols
Source: Plants (Basel). 2023 Feb 21;12(5):979. doi: 10.3390/plants12050979 (PMC10005766; doi:10.3390/plants12050979)
Supplement: Supplementary file 1 [file plants-12-00979-s001.zip › plants-2207510-supplementary.pdf]

# Holistic Photoprotection, Broad Spectrum (UVA-UVB), and Biological Effective Protection Factors (BEPFs) from *Baccharis antioquensis* Hydrolysates Polyphenols

**Yéssica A. Monsalve-Bustamante [1], Félix López Figueroa [2], Julia Vega [2], Bruna Rodrigues Moreira [3], Miguel Puertas-Mejía [1] and Juan C. Mejía-Giraldo [1,4,*]**

[1] Grupo de Investigación en Compuestos Funcionales, Facultad de Ciencias Exactas y Naturales, Universidad de Antioquia UdeA, Calle 70 No. 52-21, Medellín 050010, Colombia
[2] Departamento de Ecología y Geología, Instituto Universitario de Biotecnología y Desarrollo Azul (IBYDA), Universidad de Málaga, Campus Universitario de Teatinos s/n, 29071 Málaga, Spain
[3] Phycology Laboratory, Botany Department, Federal University of Santa Catarina, Florianopolis 88049-900, SC, Brazil
[4] Grupo de Estabilidad de Medicamentos, Cosméticos y Alimentos, Facultad de Ciencias Farmacéuticas y Alimentarias, Universidad de Antioquia UdeA, Carrera 50A No 63-85, Medellín 050010, Colombia
* Correspondence: juan.mejia8@udea.edu.co

ORCID of the author

Juan C. Mejía-Giraldo 0000-0002-2722-988X

## Supplementary Materials (SM)

## Figure S1. Extractions assay 1

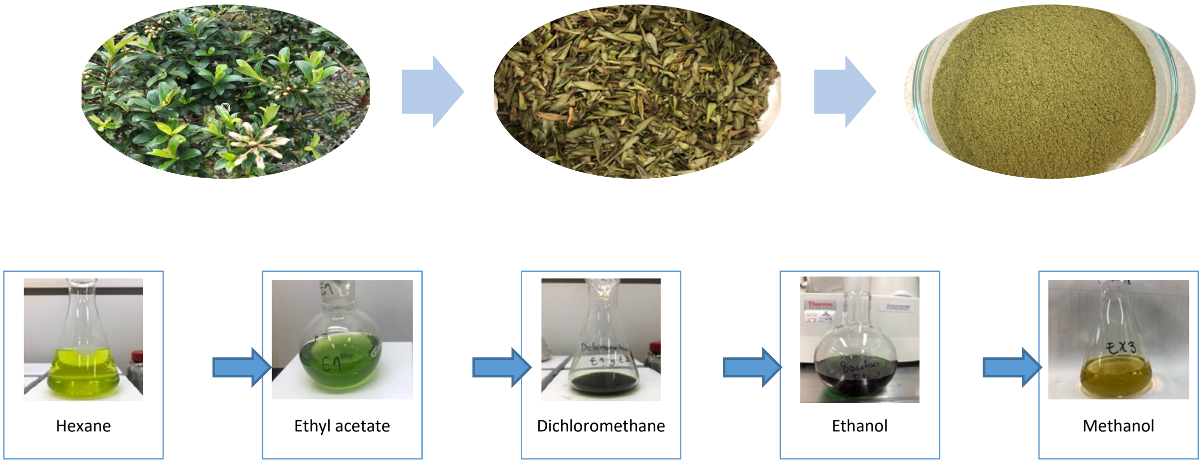


## Figure S2. Dry methanolic extract (DME) assay 3

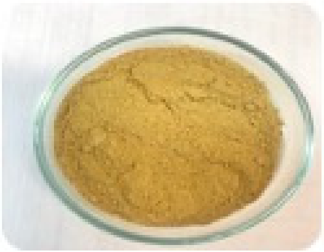

## Figure S3. Chromatographic profile of DME

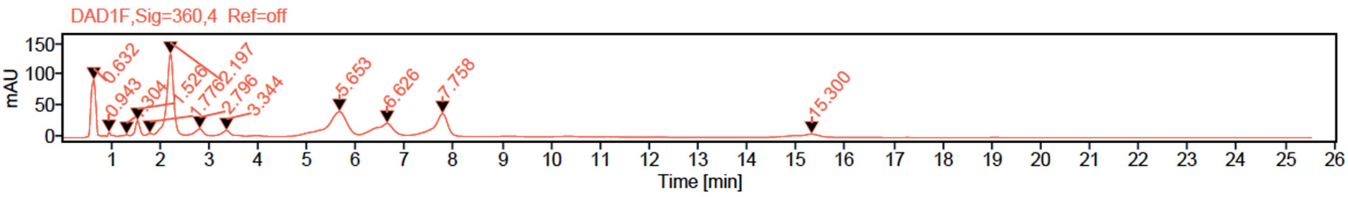

## Figure S4. UV spectrum of main DME components

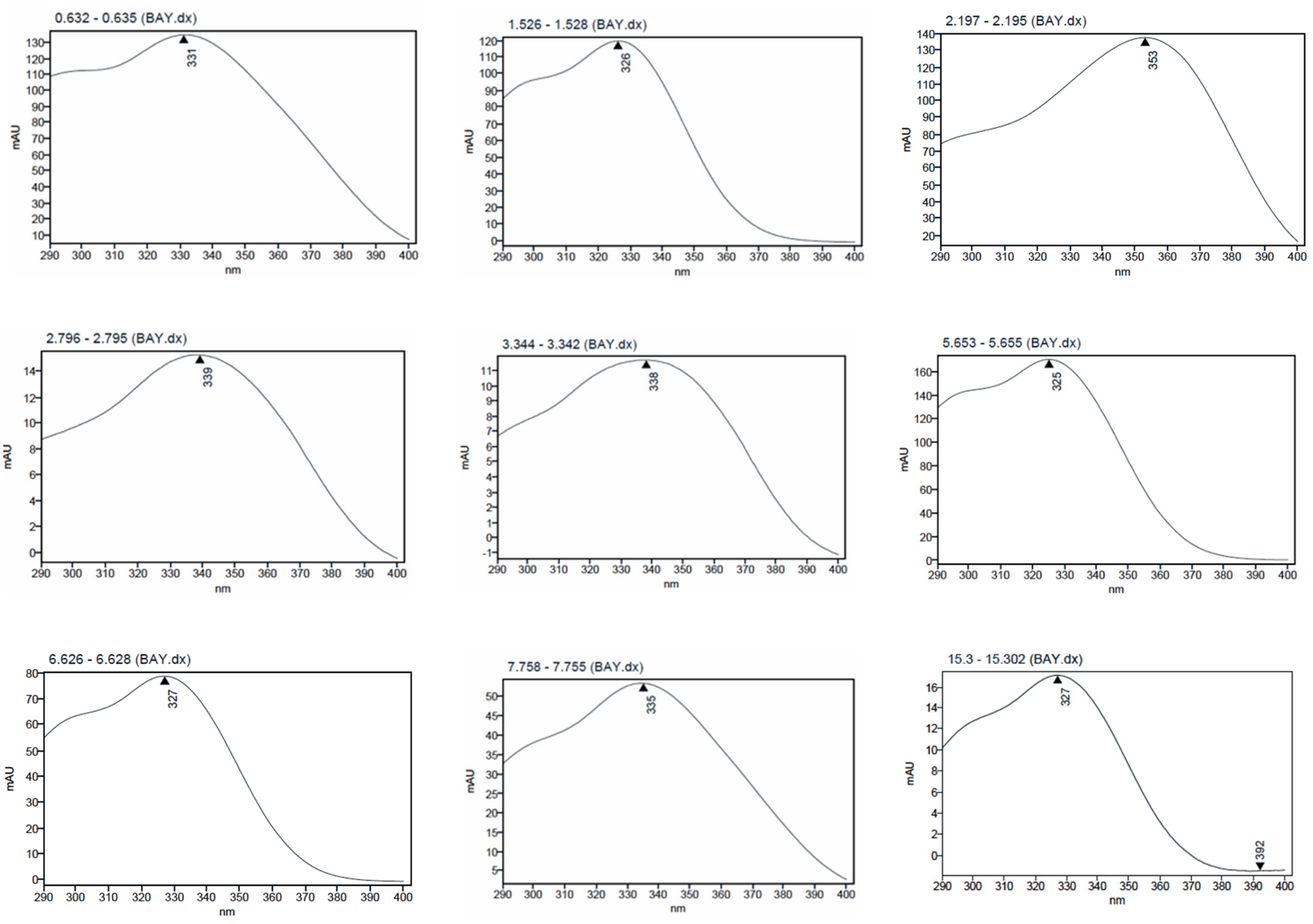


## Figure S5. Chromatographic profile of PME

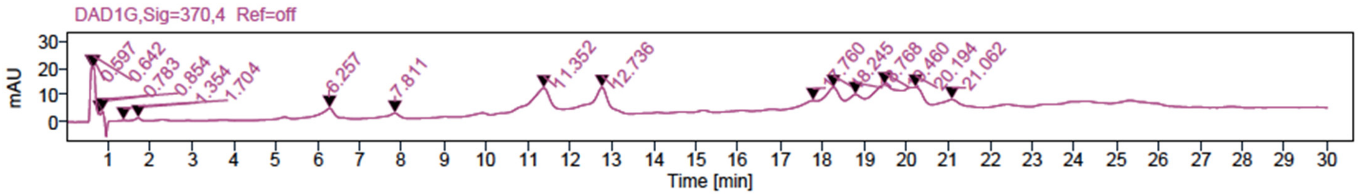

**Figure S6. UV spectrum of main PME components**

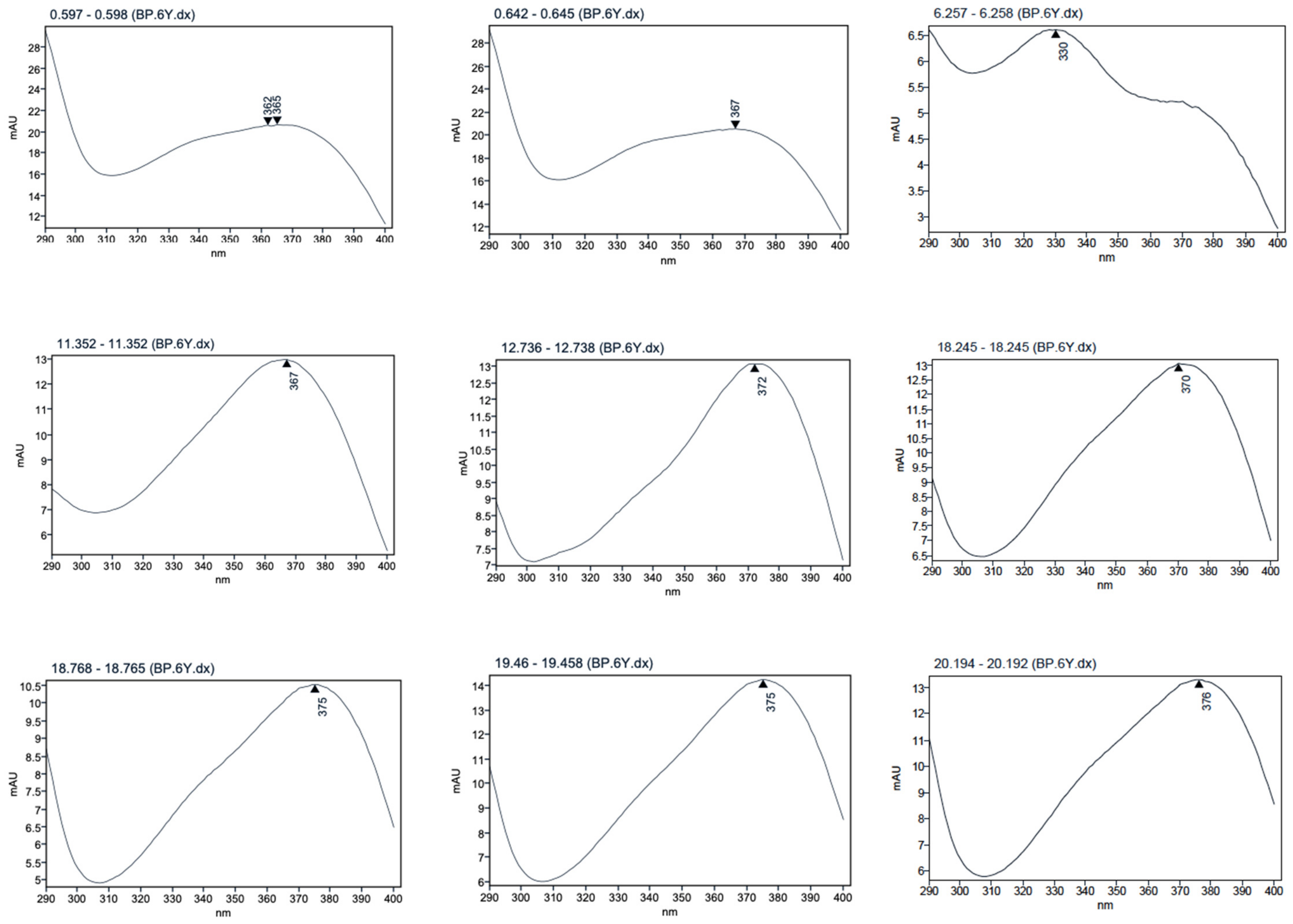

## Figure S7. Chromatograms and UV Spectra of Rutin and Quercetin

Rutin

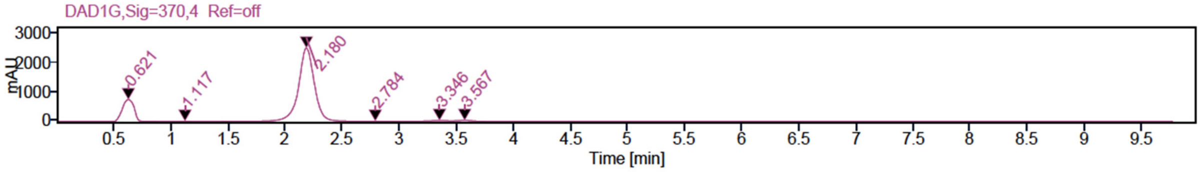


Quercetin

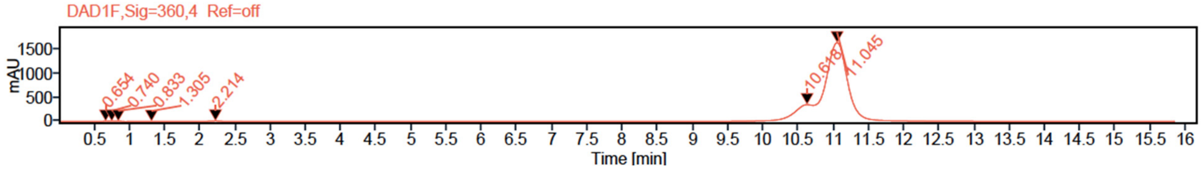


Rutin

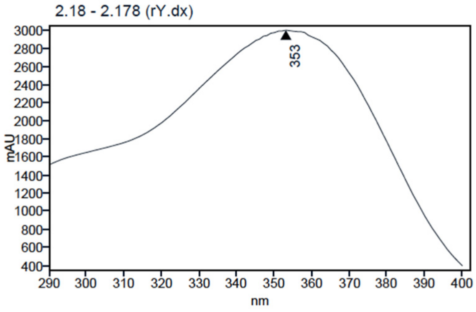


Quercetin

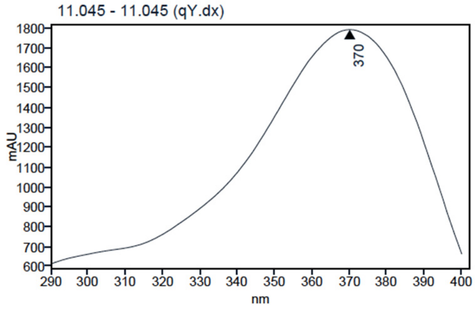


## Figure S8. ESI-MS/MS chromatogram for DME.

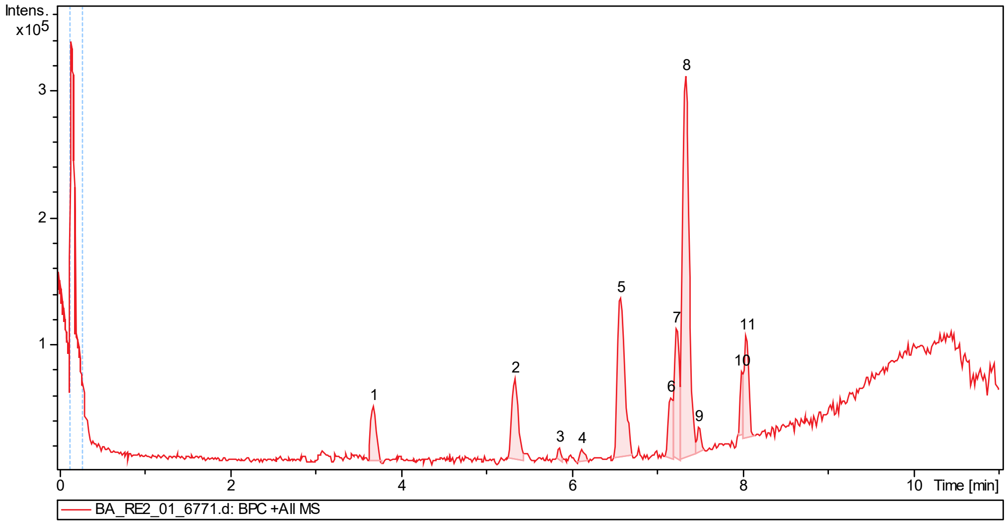

**Figure S9. ESI-MS/MS chromatogram for PME.**

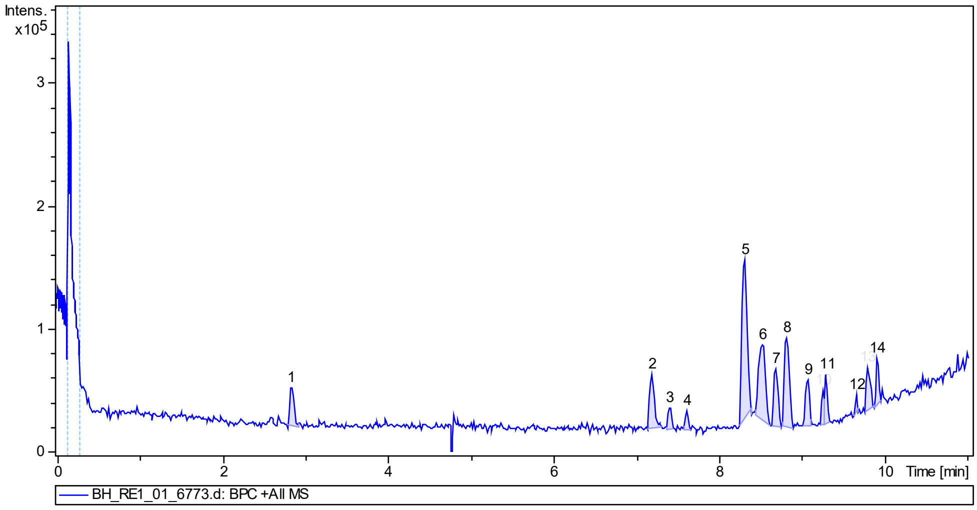


**Figure S10. Cell viability of HaCaT exposed to PME.**

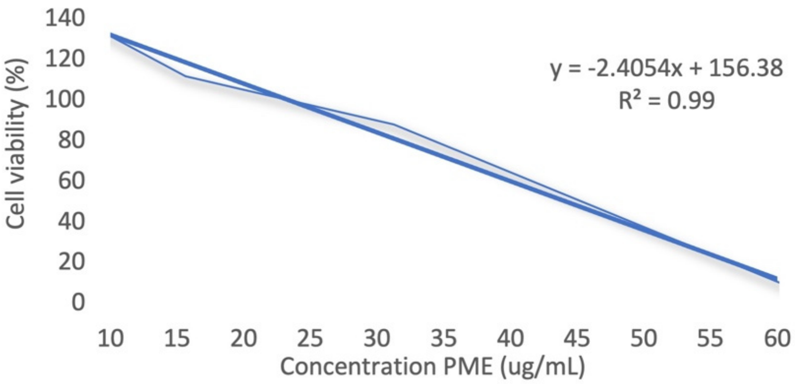

**Table S1. Spectral characteristics of the compounds identified in DME.**

| Peak | RT | Spectrum | ID |
|---|---|---|---|
| 2 | 5.4 | 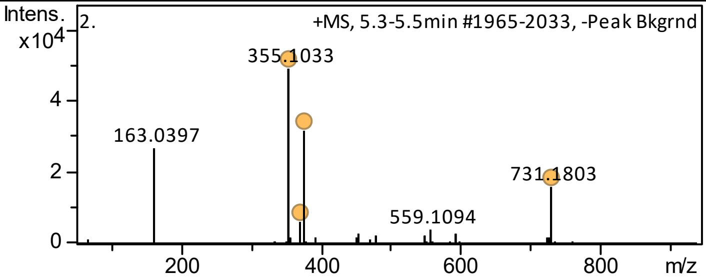  | 3-O-caffeoylquinic acid (chlorogenic acid) |
| 5 | 6.6 | 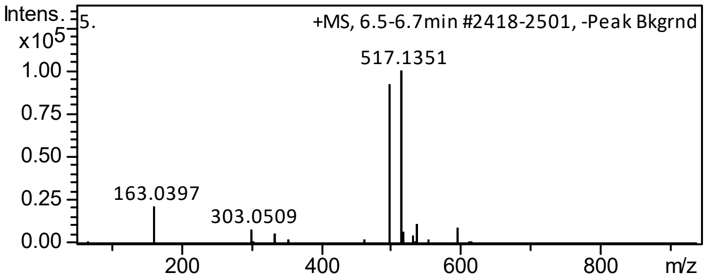  | Caffeoylquinic acid hexose |
| 7 | 7.2 | 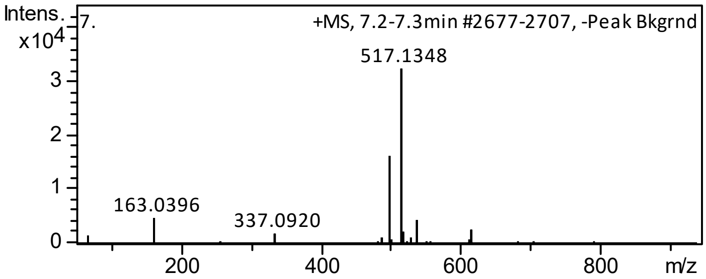  | Caffeoylquinic acid hexose |
| 8 | 7.3 | 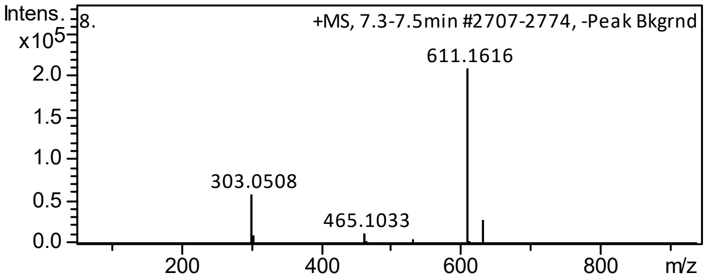  | Quercetin 3-O-rhamnopyranosyl-(1→6)-glucopyranose (rutin) |
| 10 | 8.0 | 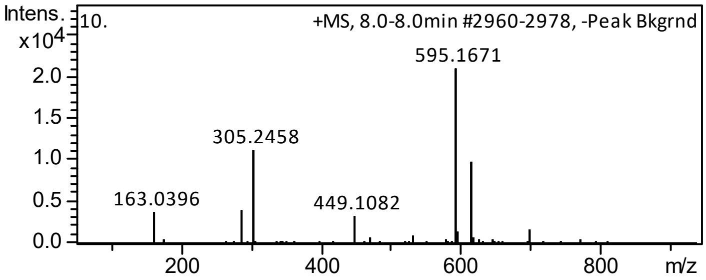  | Kaempferol 3-O-rhamnopyranosyl-(1→6)-glucopyranose |

| | | | |
|---|---|---|---|
| 11 | 8.0 | 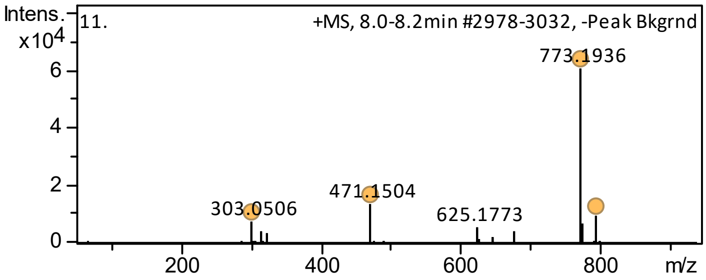 | Quercetin-3-O-(4‴-O-caffeoyl)-rhamnopyranosyl-(1→6)-galactopyranose |

**Table S2. Spectral characteristics of the compounds identified in PME.**

| Peak | RT | Spectrum | ID |
|---|---|---|---|
| 1 | 2.8 | 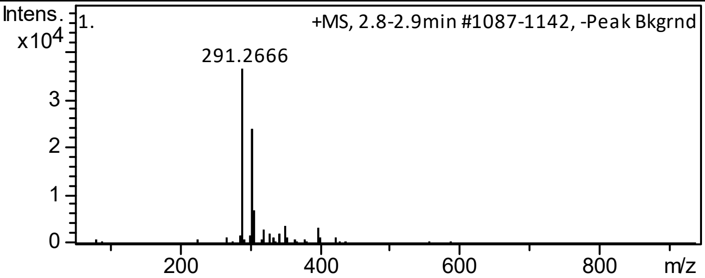  | epicatechin/catechin |
| 10 | 9.3 | 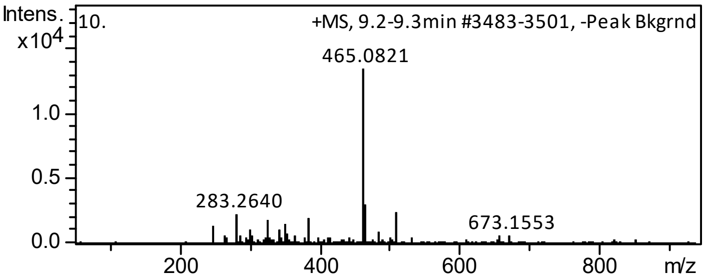  | Quercetin-3-glucoside |
| 11 | 9.3 | 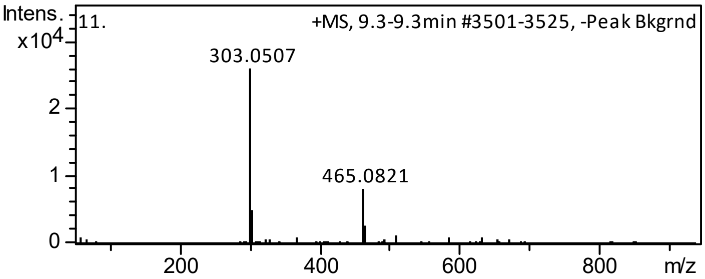  | Quercetin-3-glucoside |
| 12 | 9.7 | 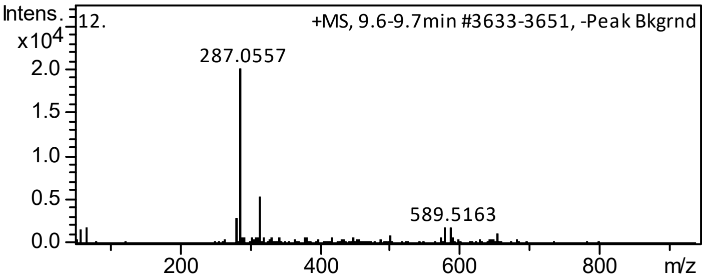  | Kaempferol |

## Table S3. Composition of the sunscreen emulsion.

| Function | Compound | Percentage in phase | Percent in emulsion |
|---|---|---|---|
| **Phase A** | | | |
| **Surfactant - emulsifying** | **Olivem 1000:** Cetearyl Olivate (and) Sorbitan Olivate | 16.0 | 4.0 |
| | **Protelan:** Glyceryl Stearate (and) Cetearyl Alcohol (and) Stearic Acid (and) Sodium Lauroyl Glutamate | 16.0 | 4.0 |
| **Emulsion stabilizing** | Stearic Acid | 4.0 | 1.0 |
| **Skin conditioning** | Sesamum Indicum Seed Oil | 12.0 | 3.0 |
| | Vitis vinifera seed oil | 8.0 | 2.0 |
| | Caprylic/Capric glycerides | 12.0 | 3.0 |
| | Mangifera Indica Seed Butte | 8.0 | 2.0 |
| | Isopropyl myristate | 8.0 | 2.0 |
| | Cetyl alcohol | 4.0 | 1.0 |
| | Cetyl palmitate | 8.0 | 2.0 |
| **Antioxidant** | Tocopherol | 4.0 | 1.0 |
| **Total in phase A** | | 100.0 | 25.0 |
| **Phase B** | | | |
| | Water | 94.9 | 56.2 |
| **Humectant** | Sorbitol 60 % | 5.1 | 3.0 |
| **Total in phase B** | | 100.0 | 59.2 |
| **Phase C** | | | |
| **Active** | Extract | 66.7 | 10.0 |
| **Solvent** | Propylene glycol | 33.3 | 5.0 |
| **Total in phase C** | | 100.0 | 15.0 |
| **Phase D** | | | |
| **Preservative** | **Geogard 221:** Dehydroacetic Acid (and) Benzyl Alcohol | 100.0 | 0.8 |
| **Total in phase D** | | 100.0 | 0.8 |
| **TOTAL** | | | **100.0** |
